# Supplementary material for: Genome-Wide Identification and Comprehensive Analysis of the GARP Transcription Factor Superfamily in Populus deltoides
Source: Genes (Basel). 2025 Mar 9;16(3):322. doi: 10.3390/genes16030322 (PMC11942272; doi:10.3390/genes16030322)
Supplement: Supplementary file 1 [file genes-16-00322-s001.zip › Figure S1.pdf]

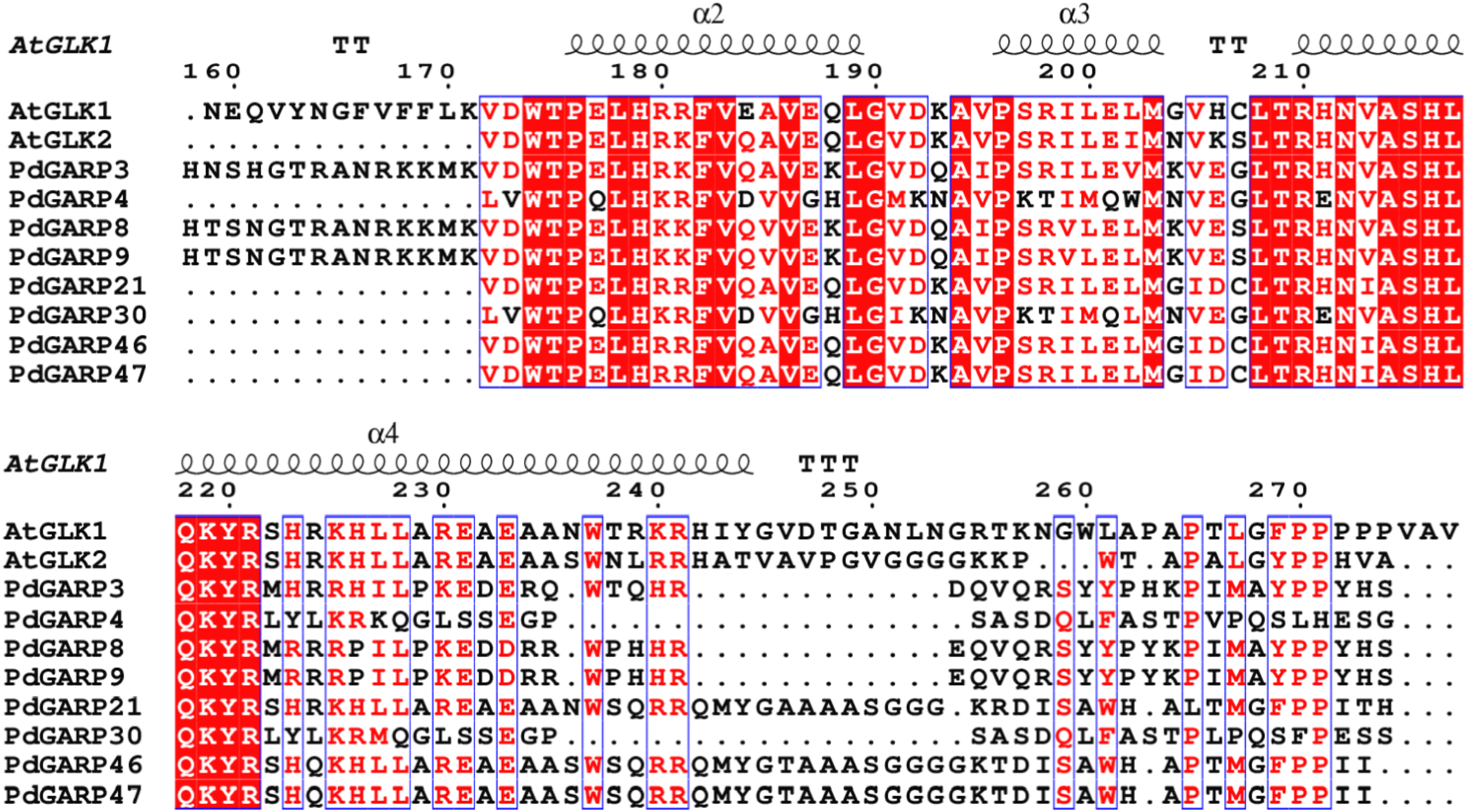
α2
α3
AtGLK1 TT TT
160 170 180 190 200 210
AtGLK1 .NEQVYNGFVFFLKVDWTPELHRRFVEAVEQLGVDKAVPSRILELMGVHCLTRHNVASHL
AtGLK2 ..............VDWTPELHRKFVQAVEQLGVDKAVPSRILEIMNVKSLTRHNVASHL
PdGARP3 HNSHGTRANRKKMKVDWTPELHRKFVQAVEKLGVDQAIPSRILEVMKVEGLTRHNVASHL
PdGARP4 ..............LVWTPQLHKRFVDVVGHLGMKNAVPKTIMQWMNVEGLTRENVASHL
PdGARP8 HTSNGTRANRKKMKVDWTPELHKKFVQVVEKLGVDQAIPSRVLELMKVESLTRHNVASHL
PdGARP9 HTSNGTRANRKKMKVDWTPELHKKFVQVVEKLGVDQAIPSRVLELMKVESLTRHNVASHL
PdGARP21 ..............VDWTPELHRRFVQAVEQLGVDKAVPSRILELMGIDCLTRHNIASHL
PdGARP30 ..............LVWTPQLHKRFVDVVGHLGIKNAVPKTIMQLMNVEGLTRENVASHL
PdGARP46 ..............VDWTPELHRRFVQAVEQLGVDKAVPSRILELMGIDCLTRHNIASHL
PdGARP47 ..............VDWTPELHRRFVQAVEQLGVDKAVPSRILELMGIDCLTRHNIASHL
α4
AtGLK1 TTT
220 230 240 250 260 270
AtGLK1 QKYRSHRKHLLAREAEAANWTRKRHIYGVDTGANLNGRTKNGWLAPAPTLGFPPPPPVAV
AtGLK2 QKYRSHRKHLLAREAEAASWNLRRHATVAVPGVGGGKKP...WT.APALGYPPHVA...
PdGARP3 QKYRMHRRHILPKEDERQ.WTQHR............DQVQRSYYPHKPIMAYPPYHS...
PdGARP4 QKYRLYLKRKQGLSSEGP..................SASDQLFASTPVPQSLHESG...
PdGARP8 QKYRMRRRPILPKEDDRR.WPHHR............EQVQRSYYPYKPIMAYPPYHS...
PdGARP9 QKYRMRRRPILPKEDDRR.WPHHR............EQVQRSYYPYKPIMAYPPYHS...
PdGARP21 QKYRSHRKHLLAREAEAANWSQRRQMYGAAAASGGG.KRDISAWH.ALTMGFPPITH...
PdGARP30 QKYRLYLKRMQGLSSEGP..................SASDQLFASTPLPQSFPESS...
PdGARP46 QKYRSHQKHLLAREAEAASWSQRRQMYGTAAASGGGGKTDISAWH.APTMGFPPII....
PdGARP47 QKYRSHQKHLLAREAEAASWSQRRQMYGTAAASGGGGKTDISAWH.APTMGFPPII....
